# Robust SrTiO_3_ Passivation of Silicon Photocathode by Reduced Graphene Oxide for Solar Water Splitting

**DOI:** 10.1021/acsami.3c07747

**Published:** 2023-09-11

**Authors:** Hsin-Chia Ho, Milutin Smiljanić, Zoran Jovanović, Miha Čekada, Janez Kovač, Gertjan Koster, Jiří Hlinka, Nejc Hodnik, Matjaž Spreitzer

**Affiliations:** † Advanced Materials Department, Jožef Stefan Institute, 1000 Ljubljana, Slovenia; ‡ Department of Materials Chemistry, National Institute of Chemistry, 1000 Ljubljana, Slovenia; § Laboratory of Physics, Vinča Institute of Nuclear Sciences—National Institute of the Republic of Serbia, University of Belgrade, 11351 Belgrade, Serbia; ∥ Department of Thin Films and Surfaces, Jožef Stefan Institute, 1000 Ljubljana, Slovenia; ⊥ Department of Surface Engineering, Jožef Stefan Institute, 1000 Ljubljana, Slovenia; ∇ MESA+ Institute for Nanotechnology, University of Twente, Enschede 7522, NB, The Netherlands; ○ Department of Dielectrics, Institute of Physics of the Czech Academy of Sciences, 182 00 Prague, Czech Republic

**Keywords:** pulsed laser deposition, SrTiO_3_, epitaxy, reduced graphene oxide, protection layer, photoelectrochemical water splitting, onset potential, stability

## Abstract

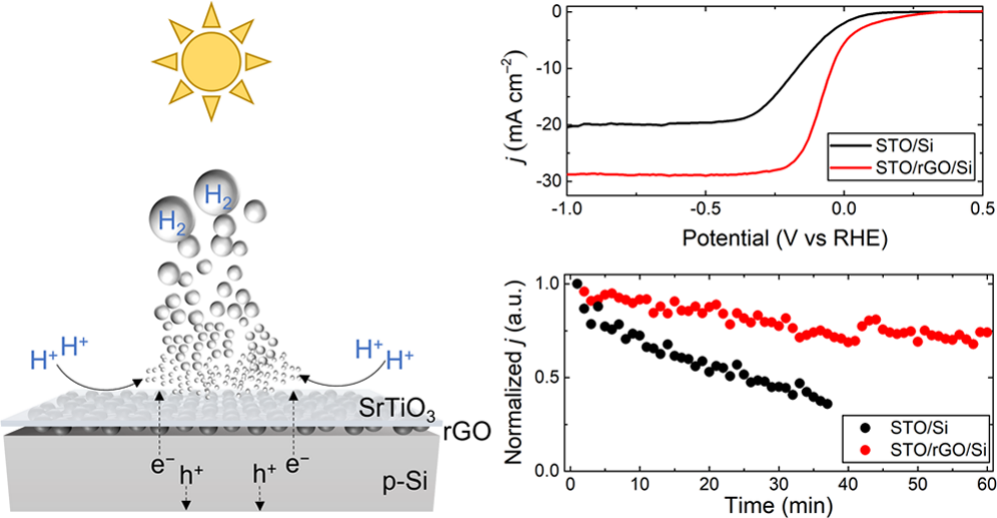

Development of a robust photocathode using low-cost and high-performing materials, e.g., p-Si, to produce clean fuel hydrogen has remained challenging since the semiconductor substrate is easily susceptible to (photo)corrosion under photoelectrochemical (PEC) operational conditions. A protective layer over the substrate to simultaneously provide corrosion resistance and maintain efficient charge transfer across the device is therefore needed. To this end, in the present work, we utilized pulsed laser deposition (PLD) to prepare a high-quality SrTiO_3_ (STO) layer to passivate the p-Si substrate using a buffer layer of reduced graphene oxide (rGO). Specifically, a very thin (3.9 nm ∼10 unit cells) STO layer epitaxially overgrown on rGO-buffered Si showed the highest onset potential (0.326 V vs RHE) in comparison to the counterparts with thicker and/or nonepitaxial STO. The photovoltage, flat-band potential, and electrochemical impedance spectroscopy measurements revealed that the epitaxial photocathode was more beneficial for charge separation, charge transfer, and targeted redox reaction than the nonepitaxial one. The STO/rGO/Si with a smooth and highly epitaxial STO layer outperforming the directly contacted STO/Si with a textured and polycrystalline STO layer showed the importance of having a well-defined passivation layer. In addition, the numerous pinholes formed in the directly contacted STO/Si led to the rapid degradation of the photocathode during the PEC measurements. The stability tests demonstrated the soundness of the epitaxial STO layer in passivating Si against corrosion. This study provided a facile approach for preparing a robust protection layer over a photoelectrode substrate in realizing an efficient and, at the same time, durable PEC device.

## 1 Introduction

With the increasing demands of sustainable energy resources in replacing fossil fuels to mitigate global warming issues, it is imperative to pursue an environmentally friendly energy-utilization approach wherein both the reactants and the products originate from clean energy sources. Photoelectrochemical (PEC) water splitting to convert water into oxygen and hydrogen gases, which principally only requires the input of solar light and water, has been regarded as one of the promising technologies to generate green hydrogen.^1^ The basic requirements of a suitable photoelectrode lie in the photoactivity of a photoabsorber producing electron/hole pairs upon illumination, the solar-to-chemical conversion efficiency, and the long-term operational stability.^2^ Various material systems have been explored as photocathodes for the hydrogen evolution reaction (HER), such as p-InP,^3^ CdTe,^4^ Cu_2_O,^5,6^ p-Si,^7−10^ to name a few. Silicon has several advantages in the HER application, including its narrow bandgap of 1.12 eV which enables the absorption of solar spectrum up to ∼1107 nm, its favorable conduction band edge alignment with respect to proton reduction potential (E(H_2_O/H_2_)) that expedites hydrogen production, and the high theoretical current densities of ∼44 mA cm^–2^ with a delivered photovoltage of 800 mV which is beneficial for decreasing the overpotential of the solar water splitting reaction.^10^ However, silicon is prone to oxidation and the intrinsic thermodynamic instability easily leads to the corrosion of silicon when in contact with an electrolyte of high ionic strength.^11^ Moreover, the high hydrogen adsorption energy on silicon surface severely impedes the proton exchange process which ultimately results in sluggish HER kinetics.^12^ Therefore, a modification of the surface by a protective layer against (photo)corrosion which at the same time could facilitate the charge transfer between the silicon photoabsorber and the electrolyte is highly desired.

Numerous materials have been developed as a passivation layer to protect p-Si photocathodes from oxidation and (photo)corrosion under the PEC operational conditions.^7,10,13−22^ Among them, transition metal oxides (e.g., TiO_2_, Al_2_O_3_, Ta_2_O_5_, etc.) have attracted great interest due to their optical transparency and robust chemical stability.^10^ When integrated with Si, these oxides with the visible light transparency allow most of the incident solar light to be harvested by the underlying Si photoabsorber, and at the same time, their chemical inertness enables the passivated photoelectrode to function well for an extended lifetime. Ta_2_O_5_ has been proposed as a viable protection material since it has a very low lattice mismatch (Si: 3.84 Å; Ta_2_O_5_: 3.80 Å)^23^ and a small conduction band offset with Si substrate,^24^ and based on that, a high-quality interface between the film and the substrate favoring charge transfer can be constructed. SrTiO_3_ (STO) is another promising candidate which exhibits a rather small compressive strain when grown on Si(001) thanks to a small (−1.7%) lattice mismatch (STO: 3.905 Å).^25,26^ The resulting epitaxial STO layer with an abrupt interface to the Si substrate could facilitate the electron/hole transportation, which also benefits from the near-zero conduction band offset.^27−29^ It was demonstrated that by conducting a strontium-assisted native oxide desorption process, unfavorable SiO_2_ that has large conduction-band offset with Si could be effectively removed, with which the photocathode exhibited a decent photocurrent density of 35 mA cm^–2^ with a remarkable onset potential shift of 450 mV and long-term stability.^30^ However, the epitaxial STO/Si photoelectrode prepared therein involved a molecular beam epitaxy (MBE) process^30^ which typically has a very low deposition rate and a high sensitivity to the deposition conditions, and therefore, a considerable difficulty in the control of reproducibility and film homogeneity is expected. Owing to this, an alternative layer deposition technique with the ease of material flux tunability (thereby the manipulation of growth speed) and simultaneously enabling the preparation of high-quality epitaxial thin films is needed if it is to be considered for industrial applications.

In the present study, we used pulsed laser deposition (PLD) to prepare an STO epitaxial layer on a Si photocathode, where in between the film and the substrate a buffer layer of reduced graphene oxide (rGO) was introduced. In our previous work we demonstrated the necessity of rGO as an epitaxy template guiding the growth of STO along the preferential orientation.^31,32^ To obtain the best quality of STO layer, an rGO-coated Si substrate with full coverage was adopted as the overgrown template.^32^ Moreover, it has been reported that the high electrical conductivity of rGO can accelerate the charge transfer between the semiconductor and the electrolyte and significantly enhance the PEC efficiency.^33,34^ Herein, the crystal and microstructural properties of the grown STO layer were investigated and discussed using X-ray diffractometry (XRD), in situ reflection high-energy electron diffraction (RHEED), and atomic force microscopy (AFM), in comparison to the polycrystalline film where STO was directly deposited on Si without rGO. The interfacial and elemental examinations revealed that a high-quality epitaxial STO layer can be readily prepared on the rGO-buffered substrate. The systematic PEC measurements and long-term stability investigation showed that with this easy fabrication approach an STO-passivated Si photocathode can remarkably enhance the PEC performance and drastically improve the stability of the Si-based photoelectrode.

## 2 Experimental Section

### 2.1 Epitaxial STO Thin-Film Deposition

Prior to the graphene oxide (GO) coating, the as-received p-Si substrate (ρ: 1–5 Ω·cm, 525 μm, Si-Mat, Germany) was ultrasonically cleaned in acetone, 2-propanol, and ultrapure water (18.2 M Ω·cm) for 15 min each. The substrate was then immersed in the heated piranha solution (3:1, 98% H_2_SO_4_: 30% H_2_O_2_, v/v) at 105 °C for 1 h followed by cooling to room temperature overnight. The purpose of the piranha treatment was to graft the hydroxyl groups on the silicon surface to transform it from a hydrophobic to hydrophilic nature, which could facilitate the uniform capturing and coating of the following GO nanosheets. Afterward, GO nanosheets obtained by centrifugation at 1000 rpm and redispersion from the as-received suspension (4 mg mL^–1^, Advanced Graphene Products, Poland) to remove agglomerated large particles, were deposited on the surface of piranha-treated Si using a spin-coating technique. The spinning speed was kept at 8000 rpm throughout the coating process while the GO suspension was applied in a dropwise fashion with the duration between each droplet (∼5 μL) of 30 s. 40 μL is required for full coverage of GO on the surface of the Si substrate.^32^ Following the spin-coating, the GO-Si sample was glued on the resistive heater by silver paste (Ted Pella, Inc.) and transferred to the UHV PLD chamber (Demcon Twente Solid State Technology, The Netherlands). The sample and the heater were degassed in ultrahigh vacuum by heating up to 750 °C and lasting for 1 h, then cooled to 650 °C for the deposition. In this way the pristine GO flakes could be mostly transformed into rGO. As for the deposition, a very thin layer of SrO (laser fluence: 2 J cm^–2^; repetition rate: 0.1 Hz; number of pulses: 15) was first deposited, followed by heating up to 755 °C to induce the deoxidation process of the Si substrate.^35^ After a few minutes, the sample was cooled to 515 °C for STO deposition. Epitaxial STO layer of a certain thickness with the deposition rate of ∼0.02 nm/pulse was then grown over the rGO-buffered Si sample in an oxygen pressure of 1.5 × 10^–6^ mbar (laser fluence: 1.5 J cm^–2^; repetition rate: 1 Hz). The same deposition procedure was carried out for the preparation of a control sample where the STO thin film was in direct contact with the Si.

### 2.2 Photoelectrode Preparation

The cocatalyst Pt was sputtered (with the effective thickness of around 0.4 nm; sputtering rate of ∼10 nm/min, using Balzers Sputron triode sputtering apparatus) on the STO/(rGO)/Si samples followed by annealing at 500 °C to form the interspersed island film. Afterward, Ga–In eutectic alloy was scratched onto the backside of the Si substrate to form an ohmic contact, and one end of the copper wire was threaded into the eutectic above which the silver paste was subsequently applied and naturally dried. Finally, the whole backside and the edges of the photoelectrode were sealed by epoxy resin where a defined surface area of the frontside was left exposed for the PEC measurements.

### 2.3 Characterization

The phase composition and crystallinity quality of the prepared samples were evaluated by means of X-ray diffractometry (XRD, Empyrean, Malvern PANalytical) in the θ–2θ scan mode with Cu Kα_1_ radiation (λ = 1.5406 Å). The in situ reflection high-energy electron diffraction (RHEED, STAIB instruments, Germany, coupled with kSA 400 RHEED analysis system from k-Space Associates, Inc., US) images were monitored and taken throughout the thin-film deposition process. The surface morphology of the samples was acquired using atomic force microscopy in the tapping mode (Veeco Dimension 3100 AFM/MFM system). The micrographs of the photoelectrode were obtained using field-emission scanning electron microscope (SEM, Thermo Fisher Verios 4G HP). The cross-sectional image of the interface was examined by transmission electron microscopy (TEM, JEOL JEM-2100) with the specimen prepared using a focused ion beam (FIB, FEI Helios Nanolab 650). The elemental depth-profiling acquisition was performed using an X-ray photoelectron spectrometer (XPS, PHI Versaprobe 3).

### 2.4 Photoelectrochemical (PEC) Measurement

The PEC measurements were conducted in a three-electrode system coupled with an electrochemical workstation (PalmSens 4, PalmSens, The Netherlands), where the prepared photocathode, platinum rod, and reference hydrogen electrode (Hydroflex, Gaskatel, Germany) were used as the working electrode, counter electrode, and reference electrode, respectively. All reported potentials are given with respect to the reversible hydrogen electrode (RHE). A solar simulator (94023A, class AAA, Newport, US) equipped with an AM 1.5 G filter was utilized as the light source where the light intensity impinging at the sample position was measured to be ∼0.8 sun using a reference silicon cell. 1 M HClO_4_, a commonly used acidic electrolyte for HER studies,^36,37^ was adopted as the electrolyte for all the PEC measurements. The linear sweep voltammetry (LSV) curves were measured from 0.5 to −1.0 V with a scan rate of 0.02 V s^–1^. The chronoamperometry where the simulated solar light chopped on and off every 60 s was performed at 0 V. The open-circuit potential was analyzed relative to RHE. Mott–Schottky (M-S) measurements were performed at a fixed frequency of 10 kHz in the dark. Electrochemical impedance spectroscopy (EIS) was acquired at 0 V over the frequency range of 10^5^–10^–1^ Hz with an AC voltage magnitude of 10 mV under solar light irradiation. Long-term stability under the constant solar illumination was tested for 1 h for samples with Pt and 8 h for sample without Pt, respectively.

## 3 Results and Discussion

### 3.1 Structural and Microstructural Properties

It is anticipated that the rGO interlayer not only can dictate the epitaxial growth of SrTiO_3_, but it can also act as an efficient conducting layer for the charge carrier transportation during photoelectrochemical (PEC) operations. Thus, intrinsically insulating GO has to be reduced to rGO in order to gain the desired conductivity.^38^ To identify the optimized parameters in obtaining rGO on Si, heat treatments ranging from 650 to 1100 °C in a UHV PLD chamber under vacuum conditions were carried out. It was observed that the carbon atoms from GO sheets readily reacted with silicon and formed the SiC_*x*_ compound accompanied by a very rough surface if the temperature was raised to 850 °C, which indicated that the heat treatment above this temperature should be avoided (see AFM images, XPS spectra, and XPS analysis in Figure S1, Figure S2, and Table S1, respectively). On the other hand, a reduced number of functional groups (epoxide and carboxyl) observed for the sample treated at 750 °C compared to 650 °C and as-prepared ones, together with nondetectable carbide signal, manifested that the optimal temperature for GO reduction on Si would be 750 °C (Figure S3). Moreover, the surface morphology generally stayed intact after 750 °C heat treatment for 1 h (Figure S1). An AFM image of the as-prepared GO-coated Si, where the Si surface was fully covered by 2–3 layers of GO sheets, can be seen in Figure S4.

**Figure 1:**
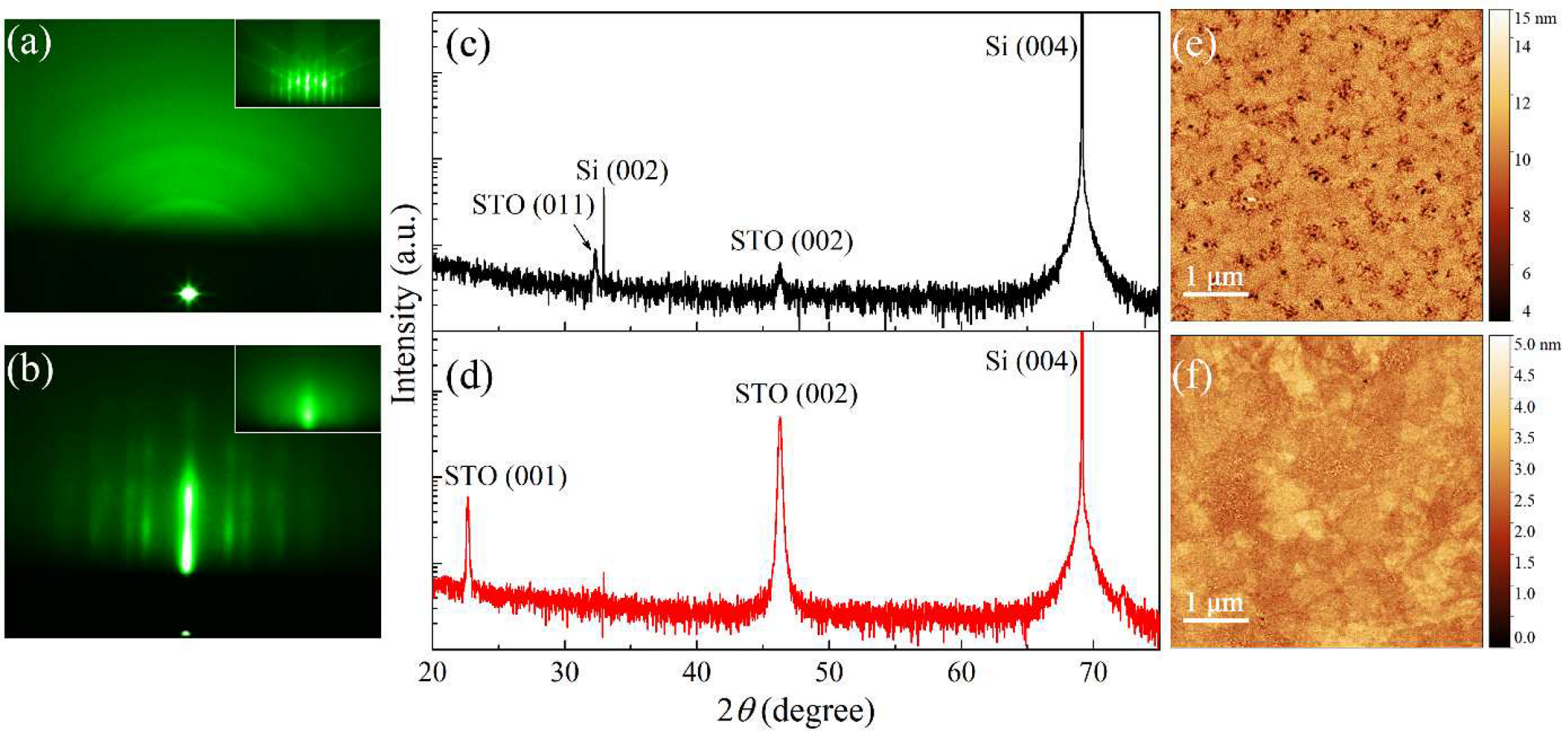
RHEED (a, b), XRD (c, d), and AFM (e, f) results of the STO thin film with a thickness of 60 nm. Top row (a, c, e): textured STO prepared on p-Si(001) substrate in the absence of rGO interlayer – STO/Si; bottom row (b, d, f): epitaxial STO deposited on rGO-buffered p-Si(001) substrate – STO/rGO/Si. Note the logarithmic scale range of *y*-axes for both XRD patterns is the same. The insets in (a, b) show the RHEED images of samples after the deoxidation step.

In order to protect the Si substrate against (photo)corrosion under the PEC operational conditions, we utilized PLD to deposit a perovskite STO layer on top of Si(001). First, the STO layer of 60 nm thickness was grown directly on the SrO-induced-deoxidized Si substrate (STO/Si). The in situ RHEED image of the as-coated STO/Si is shown in Figure 1a, where only faint ring patterns with the diffuse background were observed, indicating that the film was characteristic of a textured surface with low crystallinity. In our previous work we have verified that before the growth of STO, the deposition of SrO with a thickness of a few atomic layers is essential in catalyzing the deoxidation of native oxide and leading to the reconstruction of the silicon surface at elevated temperature, and this step can drastically improve the quality of the following deposited STO layer.^32,39^ However, despite the fact that the reconstructed Si surface was obtained after the deoxidation step (shown in the inset of Figure 1a), which can be evidenced by the narrow and sharp streaks (first-order and half-order diffractions) together with the Kikuchi lines, the oxygen element injected from the STO target could easily react with the topmost silicon atoms and lead to the formation of a silicate layer.^40^ The presence of this oxidized layer severely hindered the epitaxial growth of STO, and eventually only a textured sample was obtained. On the other hand, when STO with the same thickness was grown on rGO-buffered Si (STO/rGO/Si), well-defined streaky patterns were observed, as shown in Figure 1b. It should be noted that here, identical deposition procedures as for the preparation of STO/Si, including the SrO-induced deoxidation, were performed for STO/rGO/Si. The reconstruction of a silicon surface could hardly be identified in RHEED because the electron diffraction mainly happens at the first few layers of material, and only the broad streaks from rGO nanosheets combined with SrO thin layer were observed (see inset of Figure 1b). On the other hand, since the silicon surface was fully covered by rGO, whereupon the SrO cannot reach the native oxide and deoxidize it, no streaks related to the reconstruction could be observed. It is worth mentioning that here in order to exploit the functionality of the rGO buffer layer, fully covered rGO on Si was used for STO deposition.

The XRD results of the as-prepared STO films also show a noticeable difference: STO/Si exhibited a polycrystalline feature with poor crystallinity (Figure 1c) which is in line with the RHEED pattern (Figure 1a), while STO/rGO/Si displayed single out-of-plane orientation where the growth direction of STO solely followed the (001) orientation of Si (Figure 1d). It must be mentioned that the rGO buffer layer played two important roles during the PLD deposition process. First, it promoted the growth of a single crystalline STO layer since there is a negligible amount of dangling bonds present on the surface of rGO and accordingly the depositing STO material can more easily retain its own crystal structure. Second, rGO effectively mitigated the intermixing reaction between the STO and the Si which was observed in the STO/Si sample, and the deposited STO can grow in the preferred orientation in line with the underlying Si substrate.^32,41^ Therefore, the degree of epitaxy was significantly improved compared to the case without rGO. Moreover, no other orientations except (00l) were detected, indicating the high quality of the STO film. In addition to the notable difference in the crystallinity, the surface morphology also varied greatly between the STO/Si and the STO/rGO/Si, as shown in the AFM images in Figure 1e,f. Numerous pinholes were present in STO film (RMS = 1.10 nm), which was in direct contact with Si, whereas a very smooth layer with atomic-scale surface roughness (RMS = 0.34 nm) was observed for STO overgrown on rGO-buffered Si. The lower diffusion energy barrier of rGO compared to that of SiO_2_ enables the depositing materials to more easily migrate and rearrange on the graphene-based surface, which otherwise tend to desorb from the SiO_2_ surface given the higher diffusion barrier.^42,43^

After deposition of the STO layer, the light reflected off the sample surface gets reduced compared to bare Si in the ultraviolet, visible, and near-infrared regions (see UV–vis spectra in Figure S5). The improvement of light absorption could be helpful in terms of solar light harvesting as the planar silicon inherently reflects away a certain amount of photons which then cannot be used for the charge carrier generation. The presence of rGO interlayer seemed not to greatly change the optical property of the STO/rGO/Si, probably because the rGO buffer layer with the initial thickness of ∼2–3 nm was further reduced in thickness during the heat treatment of PLD deposition. Such a thin layer with the random distribution might not lead to a distinct alteration of light absorption.

**Figure 2:**
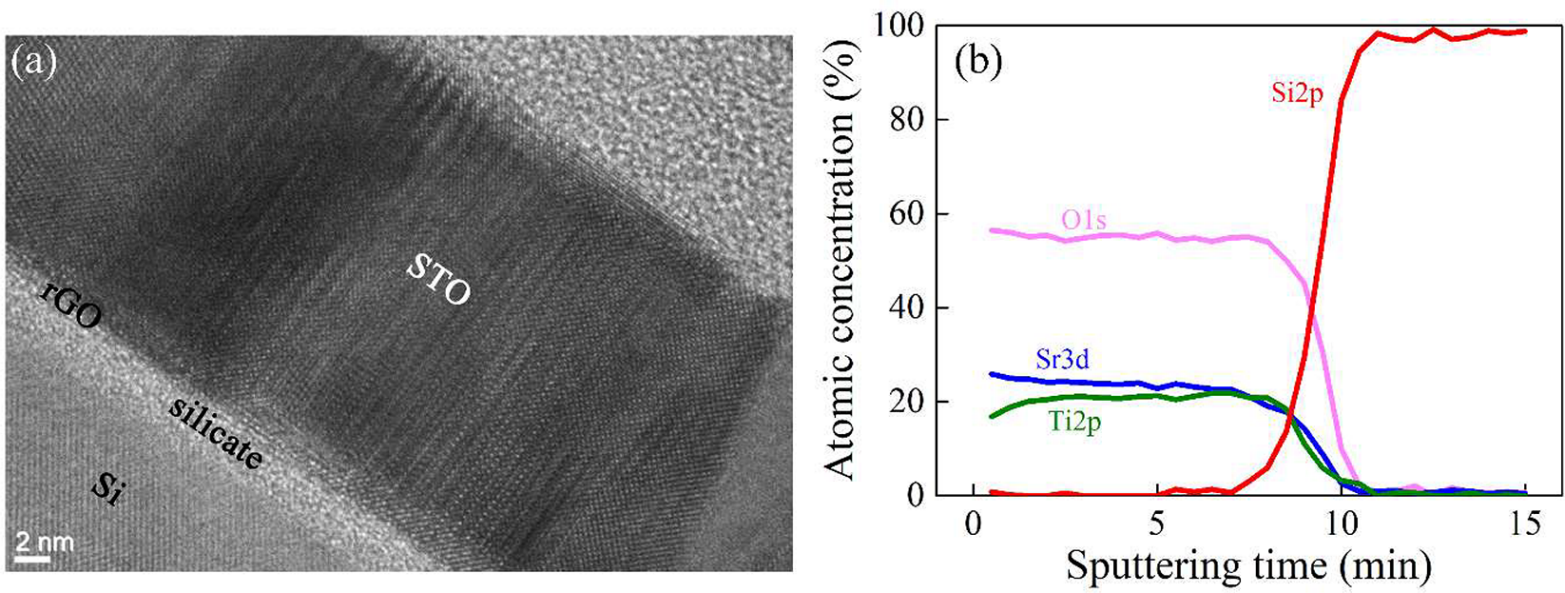
(a) TEM cross-sectional image and (b) XPS depth-profiling curves of STO (20 nm) grown on rGO-covered Si.

The cross-sectional TEM image (Figure 2a) of the epitaxial sample where the STO film was deposited upon the optimized rGO nanosheet-covered Si clearly exhibits ordered atomic arrangements across the STO layer. A region of polycrystalline STO could be seen, e.g., on the lower right part, which was not observed in the XRD θ–2θ measurement (Figure 1d) possibly due to their very small volume fraction. Interestingly, the in-plane crystal orientation relationship between STO and Si vanished (Figure S6), implying that the epitaxial relationship between the film and the substrate does not exist.^40^ In fact, the STO epitaxial registry on rGO buffer layer was favored due to <1% mismatch between the two materials,^32,44^ and that gave rise to a highly crystalline sample as evidenced from out-of-plane orientation and TEM results. The atomic arrangement diagnosed by XPS depth profiling shows that at the interface between STO and Si an intermixing reaction took place where strontium titanium silicate formed (Figure 2b), which can be correlated with the ∼2 nm amorphous layer observed from TEM. A slightly higher amount of Sr than Ti noticed in the STO layer primarily resulted from preferential ablation. During deposition the evaporation process should be taken into account rather than only the desired nonequilibrium ablation if the laser fluence was too low.^45−47^ A resputtering on the film leading to off-stoichiometry should also be considered since the deposition was performed in the vacuum regime (10^–6^ mbar of O_2_) where the plasma plume easily reached the substrate with high kinetic energy. The excess of Sr during the growth of STO layer was reported to be favorable for crystallization in the MBE system, where phase domains were separated by vertical SrO stacking faults resulting from additional Sr.^48^

### 3.2 Photoelectrochemical (PEC) Performance

**Figure 3:**
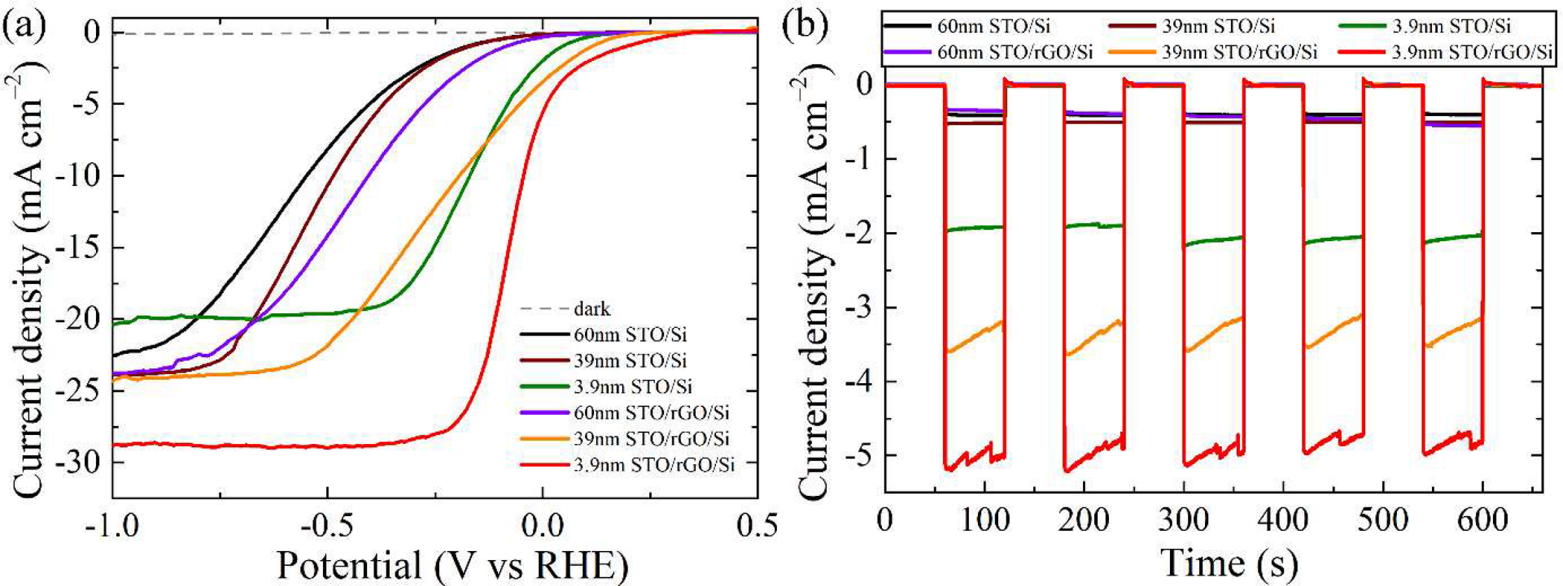
(a) Polarization curves of photocathodes with an STO layer of different thicknesses on bare Si and rGO-buffered Si. The current measured in the dark for 3.9 nm STO/rGO/Si sample is denoted as a gray dashed curve. (b) Chronoamperometry measured at 0 V (vs RHE) with illumination chopped on/off every 60 s. All samples were coated with Pt cocatalyst.

To verify the effect of the PLD-made epitaxial STO layer on improving the photoresponsive charge carrier transfer as well as on protecting the Si photocathode from corrosion during PEC operations, different thicknesses (3.9, 39, and 60 nm) of STO on rGO-buffered Si were prepared, while STO-Si directly contacted samples possessing polycrystalline and rough surface were also comparatively studied. The corresponding RHEED and AFM images of the epitaxial samples with three thicknesses can be seen in Figure S7. The transformation of Pt cocatalyst from flat film to island form was also monitored and is shown in Figure S8 and Figure S9. As shown in Figure 3a, polarization curves of the photocathode with the thickest STO (60 nm) showed a slightly lower photocurrent density with the most negative onset potential (see Table 1 for the onset value of each sample) in comparison to the thinner counterparts in both direct contact and remote epitaxy cases, implying that the photogenerated electrons from Si required a higher energy to be prompted across the STO layer to the interface between the sample and the electrolyte for the desired hydrogen evolution reaction (HER) to occur. In effect, when comparing the external energy for the cathodic reaction to start occurring, the STO/rGO/Si with the conductive rGO and high-quality STO layer would require less energy than the STO/Si, where STO was polycrystalline and the charge recombination took place before the electrons reached the interface with water. Furthermore, as evidenced in AFM image (Figure 1e), the grown STO layer with numerous pinholes in the STO/Si would very likely expose the underlying substrate to the electrolyte, which resulted in sample degradation and poor response.^49^

**Table 1:** Values Determined from LSV Measurements (Figure 3)

Sample	Onset potential^a^ (V vs RHE)	Saturation current reached^b^
60 nm STO/Si	0.033	No
39 nm STO/Si	0.036	No
3.9 nm STO/Si	0.158	Yes
60 nm STO/rGO/Si	0.089	No
39 nm STO/rGO/Si	0.216	No
3.9 nm STO/rGO/Si	0.326	Yes

a Defined as the position where current density reached −0.1 mA cm^–2^.

b Set at −0.5 V (vs RHE).

Interestingly, the onset potential was positively shifted as the STO thickness was reduced from 60 to 39 nm. In the presence of buffered layer rGO, the positive position shift of 0.127 V (from 0.089 V for 60 nm STO to 0.216 V for 39 nm STO) revealed that the HER reaction started earlier if a thinner protection layer STO was used as the transportation path for photogenerated electrons was shorter, and the incidence of energy loss and charge recombination was reduced. For the polycrystalline sample, although thinner STO indeed produced a slightly higher current density (−23.8 mA cm^–2^ for 39 nm STO vs −22.0 mA cm^–2^ for 60 nm STO), the marginal shift of onset implied that thermodynamically it was difficult for electrons to migrate across the polycrystalline STO layer given that the barrier was too high. With the thinnest protection layer of 3.9 nm, both direct contacted (STO/Si) and epitaxial (STO/rGO/Si) photocathodes exhibited the best performance compared to their respective thicker counterparts. This was primarily because the conduction channel formed by the STO layer became the shortest such that electrons could be easily extracted from the sample for HER to proceed. Despite the most positive onset position (even better than 60 nm STO/rGO/Si), 3.9 nm STO/Si delivered the lowest saturation current compared to 39 and 60 nm STO/Si, presumably because the pinholes formed during the PLD process accelerated the substrate degradation, of which a portion of the area was directly exposed to the acidic electrolyte. Notably, 3.9 nm STO/rGO/Si showed the most positive onset potential together with the highest saturation current, among all the samples studied in this work (see also Table S2 for the comparison of photocathode performance with literature values). Three synergistic effects are proposed to play the role here: (1) rGO as an efficient electron conducting layer accelerates the charge transfer;^33,34^ (2) the STO thickness is vital—if STO is too thick, the chance of the undesired recombination would be drastically increased;^30^ (3) crystallinity of the protection layer is beneficial for charge transport—the single-crystal STO layer creates an easier path for the charge transfer across the protection layer than the polycrystalline one. We tried to calculate the half-cell solar-to-hydrogen (HC-STH) efficiency, and the results are shown in Figure S10. Similarly, the 3.9 nm STO/rGO/Si sample exhibited the highest efficiency in comparison to the thicker and direct contacted STO/Si counterparts. The PEC behaviors of samples without Pt cocatalyst (Figure S11) revealed the similar trend of improvement when the buffer layer rGO was used in comparison to the directly contacted STO/Si heterostructure. It could also be noted from Figure S11 that the photoactivity of the rGO-decorated Si-based photocathode was even better than that of STO/Si, which again emphasized the important role of the conductive rGO in improving the PEC performance.

The photoresponse measured at 0 V (vs RHE) with the illumination chopped on/off is shown in Figure 3b. The generated photocurrents were consistent with the values obtained in LSV measurements. The difficulties arising from the formation of microbubbles during gas-evolving reactions, such as HER, are well-known phenomena in electrocatalysis. Attachment of the microbubbles on the active sites leads to their subsequent blockage and passivation, and this effect is difficult to mitigate.^37,50^ Therefore, the accumulation of evolved H_2_ microbubbles gradually obstructed the reaction sites between the sample and the electrolyte, as evidenced by the slow decrease of photocurrent (especially for 39 nm STO/rGO/Si and 3.9 nm STO/rGO/Si) when the samples were illuminated. Since the photoresponse upon the next cycle of illumination can return to the initial value, this means that the observed current decay was not related to the intrinsic instability of the prepared photocathodes. Because the polarization and chronoamperometry behaviors of photocathodes with 3.9 nm STO outperformed the thicker counterparts for both direct contacted and epitaxial samples, unless noted otherwise, Si photocathodes with a 3.9 nm STO protection layer were adopted for the following comparative analyses.

**Figure 4:**
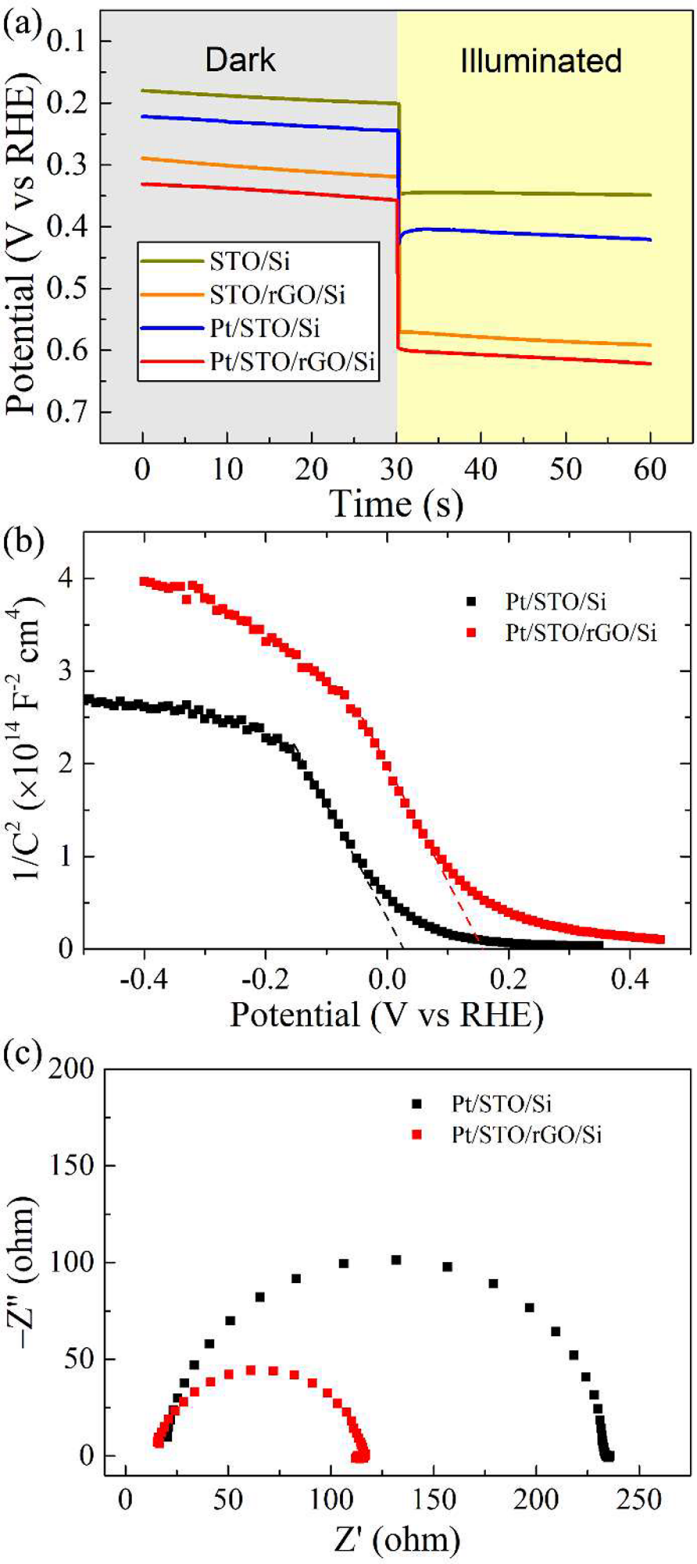
(a) Open-circuit potential measurement for 3.9 nm STO/Si, 3.9 nm STO/rGO/Si, Pt/3.9 nm STO/Si, and Pt/3.9 nm STO/rGO/Si. (b) Mott–Schottky plots for Pt/3.9 nm STO/Si and Pt/3.9 nm STO/rGO/Si. (c) Nyquist plot for Pt/3.9 nm STO/Si and Pt/3.9 nm STO/rGO/Si measured at a potential of 0 V (vs RHE) in the frequency range of 10^5^–10^–1^ Hz.

Interfacial quality between the protection layer and the substrate is important in determining the charge carrier transfer efficiency.^30,51^ The built-in field created in the photoelectrode device upon solar light illumination can be studied via open-circuit potential (*V*_oc_) measurement, where the difference between *V*_oc_ under illumination and *V*_oc_ in the dark could be translated into the photovoltage (*V*_ph_; *V*_ph_ = *V*_oc,light_ – *V*_oc,dark_).^20,51−54^ As shown in Figure 4a, without Pt cocatalyst and in the dark conditions, *V*_oc_ of STO/rGO/Si was positioned ∼0.11 V lower than that of STO/Si, indicating the difference in surface potential alignment at the front layers of photocathodes. Upon illumination, *V*_oc_ of STO/Si slightly moved down to ∼0.34 V vs RHE, representing a small *V*_ph_ of ∼0.15 V, whereas that of STO/rGO/Si increased to ∼0.58 V vs RHE, which translated to *V*_ph_ of ∼0.28 V. The generated photovoltage is basically equal to quasi-Fermi level splitting in the photoelectrode under illumination, and it signifies how efficient the charge carrier could be separated in the bulk (see Figure S12 and the Supporting Information for the discussion).^55^ The noticeable difference observed in the two photocathodes possibly resulted from the crystalline quality of the STO layer. In addition, the close contact between Si and rGO forming a Schottky junction could also help extract photogenerated electrons.^56^ Similar photovoltages were obtained after Pt cocatalyst decoration, implying the stable solid/liquid junction between the photocathode and the electrolyte. The less photovoltage obtained compared to the theoretical maximum for silicon photovoltaics of 700–800 mV might be related to the presence of a silicate interlayer, STO layer quality, and some minor structural defects.

To understand the band bending of the photocathodes, capacitance variation as a function of the applied potential without illumination was studied, and the corresponding Mott–Schottky (M-S) plots are shown in Figure 4b. The negative slope of the linear fitting indicated a p-type semiconductor characteristic. An important piece of information one can get from the M-S plot is the flat-band potential (*V*_fb_), which can be estimated by extrapolating the linear fitting to the *x*-axis.^20,52^ Here, the *V*_fb_ of Pt/STO/rGO/Si is ∼0.16 V more positive than that of Pt/STO/Si, which is in agreement with the trend in photovoltage measurements. As suggested by the equation of *V*_b_ = *V*_a_ – *V*_fb_ (*V*_b_ is the band bending, *V*_a_ is the applied potential),^57^ the positive shift of *V*_fb_ would give rise to a larger band bending at the interface between the photoelectrode and the electrolyte, and that in turn could simultaneously enhance the charge separation and suppress the charge recombination.

In the photocathode, after the photogenerated electrons are separated from the holes and transported to the electrode surface, the resistance of the charge carrier transfer at the interface between the photocathode and the electrolyte would be decisive in interpreting the efficiency of the HER reaction. To elucidate this, EIS spectra were measured, and the Nyquist plots are shown in Figure 4c (see Figure S13 for EIS measurements of samples without Pt cocatalyst). In general, a smaller semicircle radius means a faster charge carrier transfer. It was found that STO with a substantial improved crystallinity resulted in a smaller arc, implying that the charge carriers were more dynamic at the interface with the electrolyte in the STO/rGO/Si than in the STO/Si. In addition, highly conductive rGO to promote electron migration between the Si and the STO could also contribute to a higher charge transfer efficiency.^34,56^ The larger arc in impedance of the STO/Si photocathode was partly attributed to the pinholes in the STO layer that interfered with the electron migration. The nonepitaxial polycrystalline STO would also reduce the charge transfer efficiency, which reemphasized the importance of an epitaxial protection layer in the photoelectrode preparation.

### 3.3 Stability Testing

**Figure 5:**
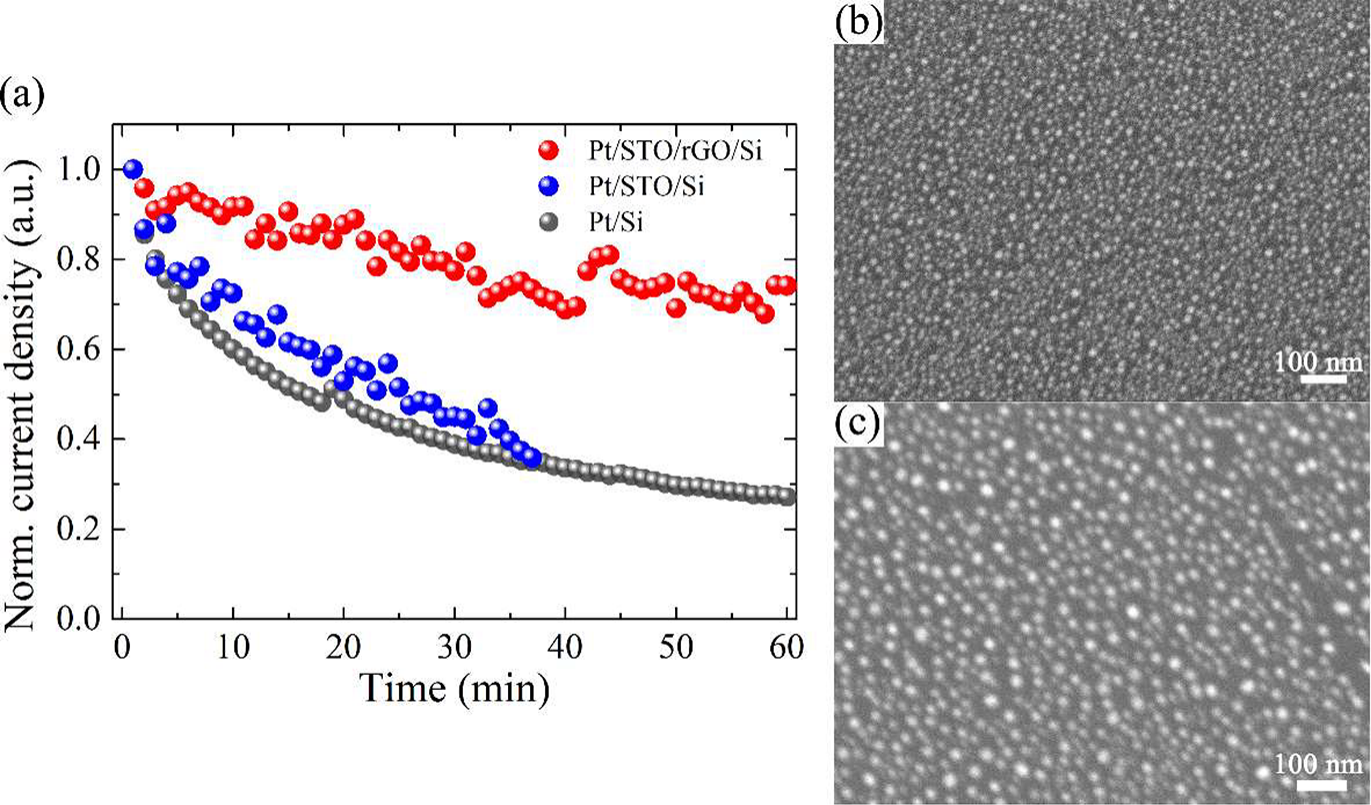
Long-term stability tests of STO passivated Si-photocathodes. (a) Chronoamperometry measurement at 0 V (vs RHE) with constant solar illumination. Note that the current densities were normalized to the initial values for the respective electrodes. SEM images of Pt/STO/rGO/Si (b) before and (c) after the long-term stability test.

The efficiency of the STO thin film, especially the epitaxially grown STO layer over the rGO mediator, in protecting silicon photocathodes from corrosion during PEC operational conditions was studied in chronoamperometric measurements at 0 V (vs RHE) under constant solar illumination. As shown in Figure 5a, for the bare Si without any protection, the photocurrent quickly dropped to below half of the initial value in 15 min. The rapid formation of an oxidized layer on the silicon surface adversely hampered the charge transfer at the interface, and as a result of this inevitable corrosion, the photoresponse decayed to below 30% after 1 h of testing. Upon direct encapsulation of the STO layer (STO/Si), the photocurrent decreased at an almost identical rate as the bare Si sample. Such fast degradation of the Si-based photocathode might be a consequence of the pinholes formed in the STO layer during PLD deposition, as observed in the surface morphology shown in Figure 1e. A great number of pinholes present in the STO/Si enabled the electrolyte to penetrate through the STO layer and to corrode the underlying substrate, which ultimately exhibited a behavior similar to that of Si without protection. On the other hand, the decay of PEC performance was substantially alleviated when the Si was passivated by an epitaxially grown STO protective layer. rGO as a buffered layer offered STO materials more time to diffuse over its surface during thin-film growth and rendered the 2D layer formation without morphological defects, as evidenced in Figure 1f. This could explain the maintenance of an efficient photoresponse of the STO/rGO/Si photocathode during the whole operation. It is worth mentioning that the noisy fluctuations in the reported data of the STO-protected photocathodes were due to the above-mentioned inevitable issue of the accumulation of H_2_ bubbles on the surface which obstructed the active sites for charge transfer, which is a well-known phenomenon encountered in gas-evolving reactions.^37,50^ Moreover, such a blockage of the active sites may contribute to the observed activity decay, meaning that it does not come from the intrinsic degradation. The same issue is much less pronounced for bare Si because the reaction was sluggish with a very small photocurrent and therefore negligible bubble generation, and the curve was much less noisy. The evolution of charge transfer resistance subjected to a long-term stability test was analyzed with impedance measurement (Figure S14). A slight enlargement of the semicircle for the STO/rGO/Si photocathode after 1 h of testing (Figure S14a) corresponded to the photocurrent decay observed in Figure 5a. Conversely, a drastic increase in arc for bare Si (Figure S14b) after the durability measurement demonstrated its susceptibility to corrosion. The stability was also compared in potentiodynamic conditions as samples were subjected to 2000 cyclic voltammetry (CV) scans. The results (Figure S15) again manifested the protection capability of the epitaxial STO layer against corrosion of the underlying substrate with only a slight increment of resistance after testing in comparison to the noticeable deterioration of the performance of the bare Si.

The exploration of morphology before and after the 1 h continuous PEC operations could possibly reveal the sample evolution taking place on the surface and the origins of the observed decay of the photoactivity. In the STO/rGO/Si photocathode, the surface generally stayed intact without any noticeable change (e.g., film delamination), as shown in Figure 5b,c. Interestingly, the Pt cocatalyst particles slightly enlarged in size from the initial 11 ± 5 nm to 17 ± 8 nm after the test. In general, the degradation of nanoparticulated Pt catalysts during HER occurs due to the migration of Pt particles over support resulting in detachment and/or particle size growth due to the coalescence.^37,58,59^ The degradation induced during long-term PEC operations resulted in larger Pt nanoparticles due to the particle agglomeration, which would parasitically reduce the light absorption of the underlying semiconductor substrate and, in turn, reduce the generated photocurrent (see the UV–vis spectrum of the sample after the PEC test in Figure S5). In addition, some Pt particles may be detached from the STO/rGO/Si surface, which would contribute to the observed activity decay. Overall, the PEC performance of STO/rGO/Si tended to stay relatively stable (Figure 5a), revealing the stability of the epitaxially STO-protected Si photocathode.

**Figure 6:**
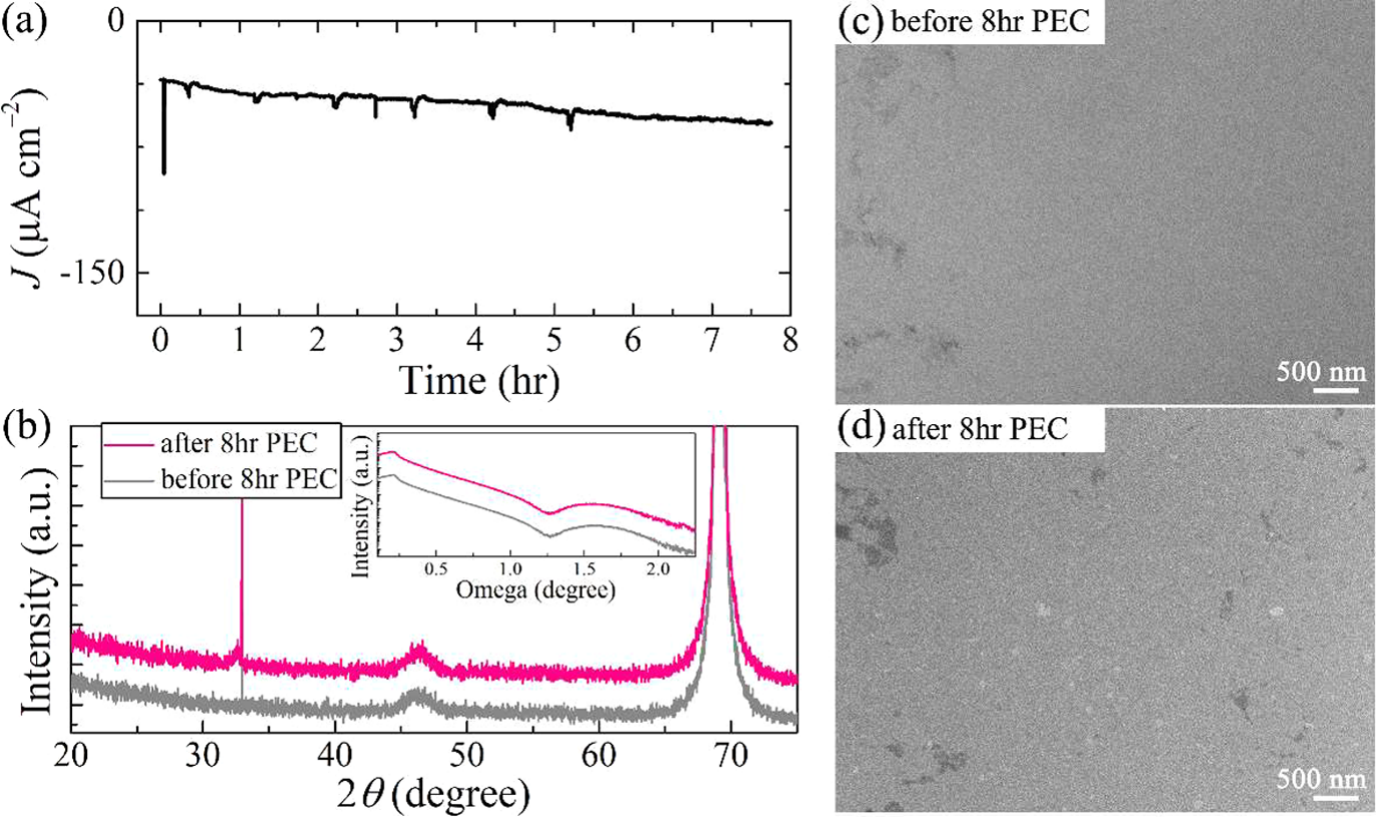
Stability test of 8 h for 3.9 nm STO/rGO/Si, without Pt cocatalyst. (a) Photocurrent measurement 0 V (vs RHE) with constant solar illumination. (b) XRD θ–2θ and XRR (inset) measurements and SEM (c, d) comparison of sample before and after 8 h test. The spikes shown in panel a are related to water-bath exchange, which is used for maintaining a constant temperature of the electrolyte solution. The diffractograms in panel b and reflectograms (b, inset) are offset for clarity. The dim regions in SEM images (c, d) come from the traces of rGO buffer layer beneath the 3.9 nm STO top layer.

To further clarify the cause of degradation, the durability of the STO/rGO/Si photocathode without Pt was tested for 8 h. It is shown that the photocurrent stayed fairly stable without a noticeable decay that is otherwise observed in the case with Pt (Figure 6a). In comparison to the initial state, the XRD results (Figure 6b) show that after 8 h of PEC operation, the predominant STO (002) peak (2θ ≈ 46.3°) appearing at the similar diffraction angle position has the comparable intensity, implying that the crystallinity of the passivated STO layer does not change. It is also noted that the quality of interfaces between STO, rGO, and Si was not affected, as could be appreciated from the X-ray reflectivity (XRR) results shown in the inset of Figure 6b. The similar critical angle positions as well as the similar reflectivity decreasing patterns manifest that the layer thickness, surface/interface roughness, and the interface properties were not altered. Furthermore, SEM was conducted to provide a direct assessment of the integrity of the sample surface being exposed to a photoelectrochemical environment. As one can see from Figure 6c,d, after the 8 h long PEC test the sample remains as intact as the initial condition, where no observable structural variation (e.g., film cracking and/or delamination) could be detected. Based on the above results, it could be argued that the PEC degradation was very likely due to the agglomeration and/or detachment of Pt during the HER process, and the degradation of the underlying STO/rGO/Si substrate could be ruled out.

## Conclusion

In this work, we demonstrated that the SrTiO_3_ (STO) layer overgrown on the Si substrate which was fully covered with a reduced graphene oxide (rGO) buffer layer could promote the charge carrier transfer across the photocathode and lead to a much better photoelectrochemical (PEC) performance. Single-oriented STO realized by rGO-buffered substrate rendered an easier path for charge migration, and the thinnest STO layer delivered the shortest transportation route while still maintaining the robust protection ability. However, polycrystalline STO in direct contact with Si presented more recombination sites which eventually impaired the PEC performance. The smooth surface of the high-quality epitaxial STO layer with a subnano roughness was demonstrated to be a pivotal factor in remotely protecting the underlying substrate from corrosion, while the presence of pinholes in the nonepitaxial sample showed a similar degradation rate as bare substrate without any protection. This study provided a detailed description and explanation of a relatively facile approach in preparing an efficient and stable passivated Si-based photocathode, which could greatly aid in the applications of protected photoelectrodes in PEC water splitting reactions.

## Supporting Information Available

Tables for XPS analysis of GO/Si samples treated at different temperatures and comparison of our photocathode’s performance with literature values; AFM images of GO/Si samples treated at different temperatures; XPS spectra of GO/Si samples treated at different temperatures; RHEED images of GO/Si; AFM image of as-prepared GO/Si; UV–vis spectra; phi-scan of STO/rGO/Si; RHEED and AFM of STO/rGO/Si with different STO thicknesses; RHEED images of platinized sample; SEM images of platinized sample; HC-STH, LSV of photocathodes without Pt; schematic mechanism; EIS spectra of photocathodes without Pt; EIS comparison before and after 1 h stability test; stability test under 2000 CV scans (PDF)
