# Correction to “Robust SrTiO_3_ Passivation of Silicon Photocathode by Reduced Graphene Oxide for Solar Water Splitting”

**DOI:** 10.1021/acsami.5c10225

**Published:** 2025-06-22

**Authors:** Hsin-Chia Ho, Milutin Smiljanić, Zoran Jovanović, Miha Čekada, Janez Kovač, Gertjan Koster, Jiří Hlinka, Nejc Hodnik, Matjaž Spreitzer

In the original version of our paper, two additional Slovenian Research Agency projects (N2-0176, P2-0091) were missed in the Acknowledgment section. In the revised version shown below, the two projects are added. The conclusions of the work are not altered.

The work was supported by the Slovenian Research Agency (Project No. N2-0187, N2-0176, P2-0091), the Czech Science Foundation 707 (Project No. 21-20110K), and the Slovenia–Serbia bilateral collaboration (Project “Photoelectrochemical Hydrogen Evolution from Epitaxial Silicon-Oxide Heterostructures (H2EPI)”). The authors also acknowledge support from Mr. Damjan Vengust for the electron microscopy observations, Mr. Blaž Jaklič for the XPS analysis, Mr. Jožko Fišer for the Pt thin-film preparation, Dr. Vasko Jovanovski for the discussion of electrochemical measurements, and Dr. Špela Kunej for the UV–vis spectroscopy measurement.

