# Supplementary material for: Robust SrTiO3 Passivation of Silicon Photocathode by Reduced Graphene Oxide for Solar Water Splitting
Source: ACS Appl Mater Interfaces. 2023 Sep 11;15(37):44482–92. doi: 10.1021/acsami.3c07747 (PMC10520914; doi:10.1021/acsami.3c07747)
Supplement: Supplementary file 1 — am3c07747_si_001.pdf [file am3c07747_si_001.pdf]

Supporting Information

Robust $SrTiO_3$ Passivation of Silicon Photocathode by Reduced Graphene Oxide for Solar Water Splitting

Hsin-Chia Ho[*,1], *Milutin Smiljanić*[2], *Zoran Jovanović*[3,1], *Miha Čekada*[4], *Janez Kovač*[5], *Gertjan Koster*[6], *Jiří Hlinka*[7], *Nejc Hodnik*[2], *and Matjaž Spreitzer*[*,1]

[1] *Advanced Materials Department, Jožef Stefan Institute, 1000 Ljubljana, Slovenia*

[2] *Department of Materials Chemistry, National Institute of Chemistry, 1000 Ljubljana, Slovenia*

[3] *Laboratory of Physics, Vinča Institute of Nuclear Sciences—National Institute of the Republic of Serbia, University of Belgrade, 11351 Belgrade, Serbia*

[4] *Department of Thin Films and Surfaces, Jožef Stefan Institute, 1000 Ljubljana, Slovenia*

[5] *Department of Surface Engineering, Jožef Stefan Institute, 1000 Ljubljana, Slovenia*

[6] *MESA+ Institute for Nanotechnology, University of Twente, 7522 NB Enschede, The Netherlands*

[7] *Department of Dielectrics, Institute of Physics of the Czech Academy of Sciences, 182 00 Prague, Czech Republic*

[*] Corresponding author

E-mail address: hsin-chia.ho@ijs.si; matjaz.spreitzer@ijs.si

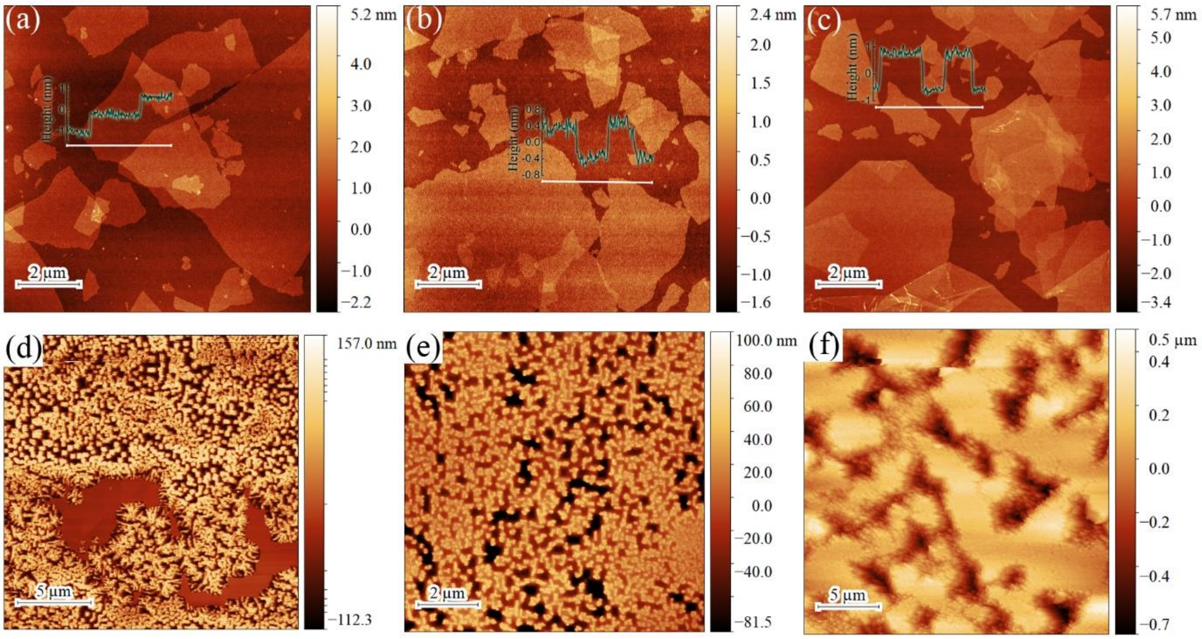

Figure S1. AFM images of GO-covered Si samples (a) as-coated, and subjected to heat treatments at (b) 650, (c) 750, (d) 850, (e) 950, and (f) 1100 °C for 1 hr in vacuum. The insets in (a-c) show the line profiles corresponding to the white line drawn over layer(s) of (r)GO sheets. Note that all the samples shown here were with half coverage of GO layers on Si surface and not used for the STO deposition.

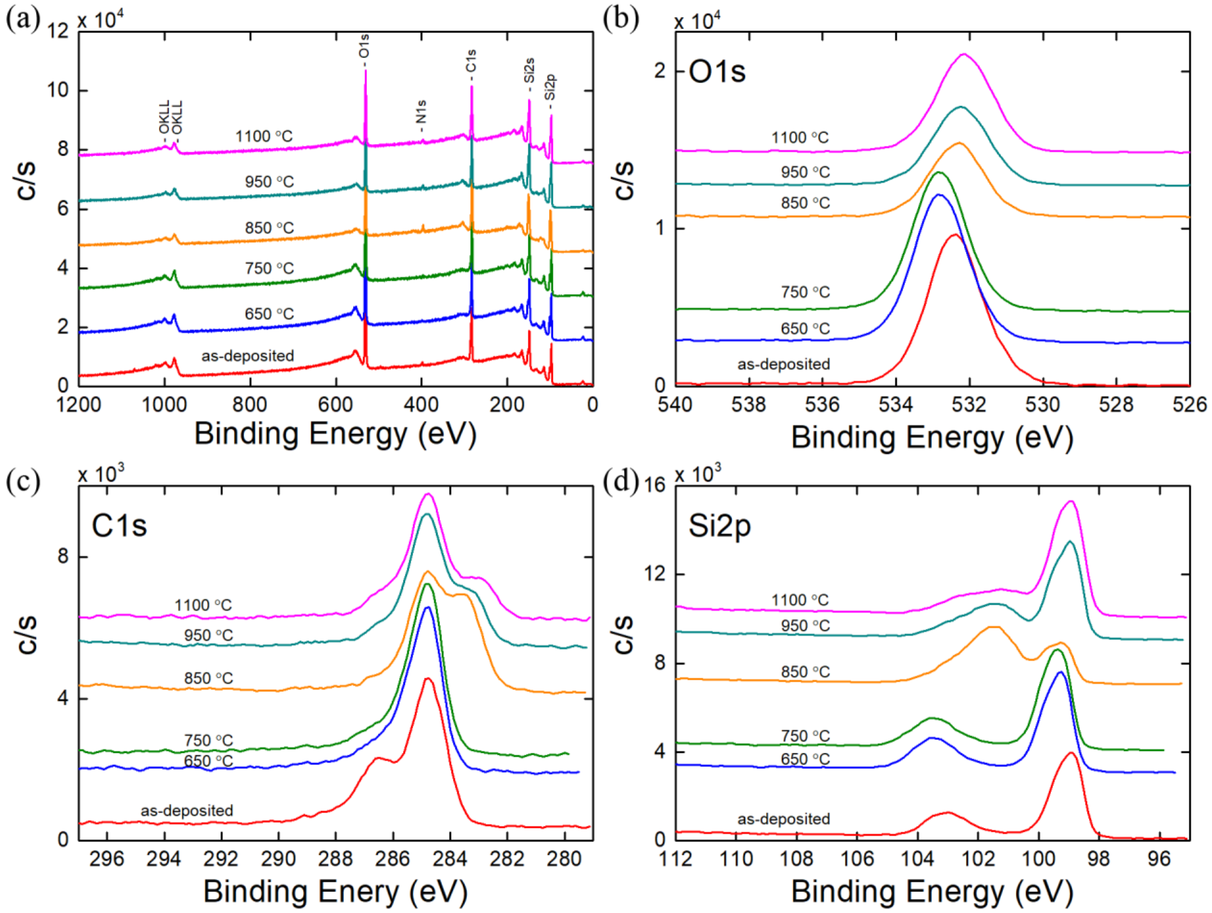

Figure S2. XPS analysis of GO-covered Si samples subject to heat treatment at different temperatures. (a) survey; (b) O 1s; (c) C 1s; and (d) Si 2p spectra.

Table S1. XPS analysis of relative atomic concentration in % of fitted peaks in C 1s and Si 2p spectra.

Component	C=C/C−C	C−O	O=C−O	carbide	Si-bulk	Si-bulk	Si−O_x	SiO_2	SiC_x
Binding energy (eV)	**284.8**	**286.0**	**288.7**	**283.3**	**98.89**	**99.50**	**100.8**	**103.0**	**101.4**
**25 °C***	61.0	32.1	6.9	0	45.8	22.9	4.8	26.5	0
**650 °C**	65.0	32.7	2.3	0	43.2	21.6	6.1	29.1	0
**750 °C**	71.3	27.0	1.7	0	43.2	21.6	6.7	28.5	0
**850 °C**	47.4	9.8	1.8	41.0	17.8	8.9	0	19.5	53.8
**950 °C**	56.1	18.6	1.5	23.8	38.6	19.3	0	10.4	31.7
**1100 °C**	55.9	18.3	1.4	24.4	42.2	21.1	0	5.9	30.8

* As-coated GO/Si sample.

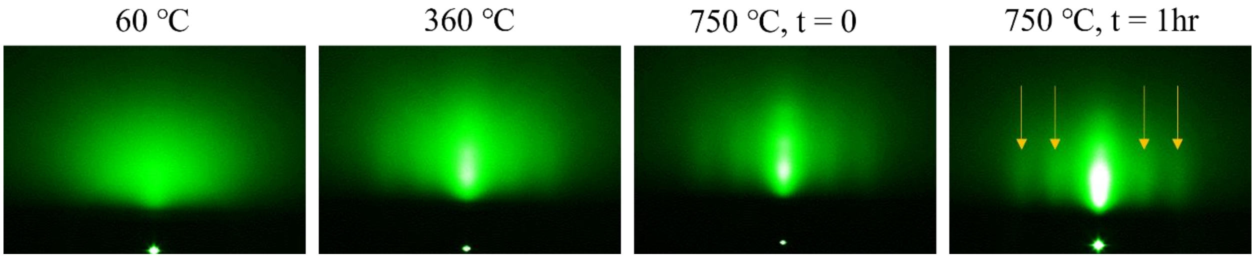

Figure S3. RHEED images of the GO-coated Si evolved from low-temperature to the optimal heat treatment temperature of 750 °C for 1hr where optimal rGO was obtained. The yellow arrows denoted the characteristic patterns of rGO.

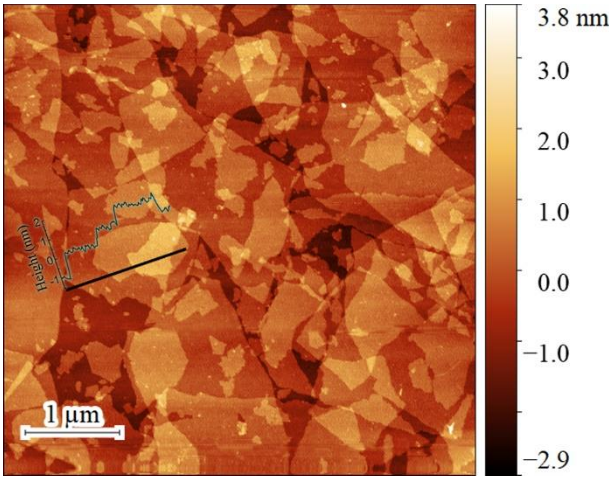

Figure S4. AFM image of the as-prepared GO/Si sample. The inset shows the line profile along the black line drawn over layers of GO sheets. Note that the STO/rGO/Si samples discussed in the main text were all prepared from the substrates with full coverage of rGO sheets.

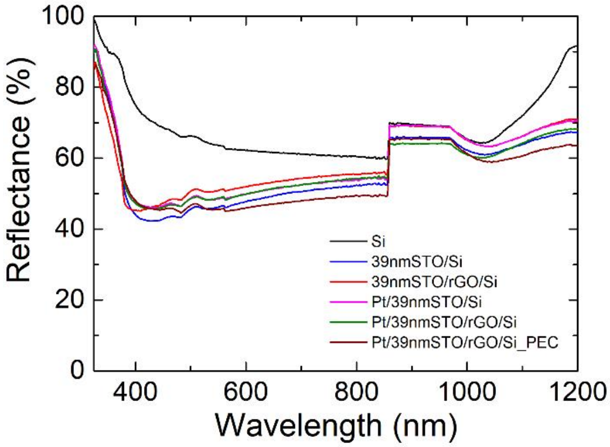

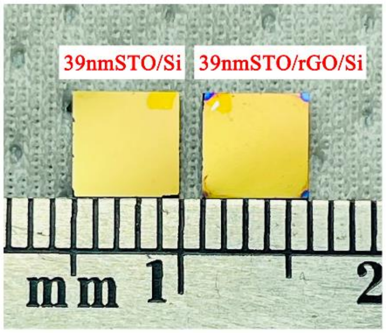

Figure S5. (Left) UV-vis spectra of bare Si, STO-protected Si with and without cocatalyst Pt, and sample after PEC measurements. The curve discontinuity at ~860 nm is resulted from the detector change in the UV-vis spectrophotometer. (Right) Photo of STO/Si and STO/rGO/Si samples. The corners of STO/rGO/Si sample with the blueish hue are from the accumulation of rGO sheets formed during the spin-coating, whereas the majority of sample surface is with ~2-3 layers of rGO and shows very similar appearance as the STO/Si sample.

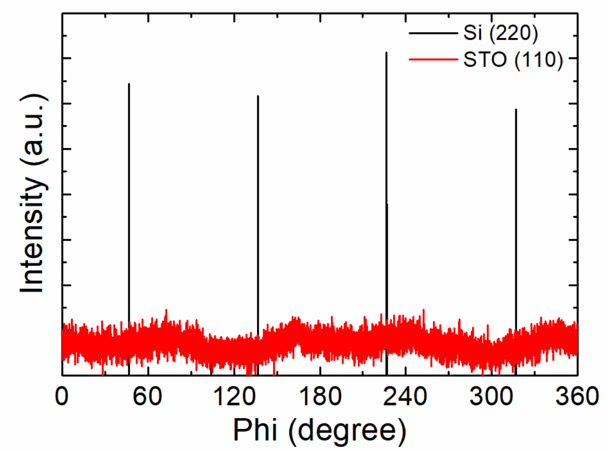

Figure S6. Phi-scan analysis of (220) and (110) peaks of the Si substrate and the STO thin film from STO (60 nm)/rGO/Si sample.

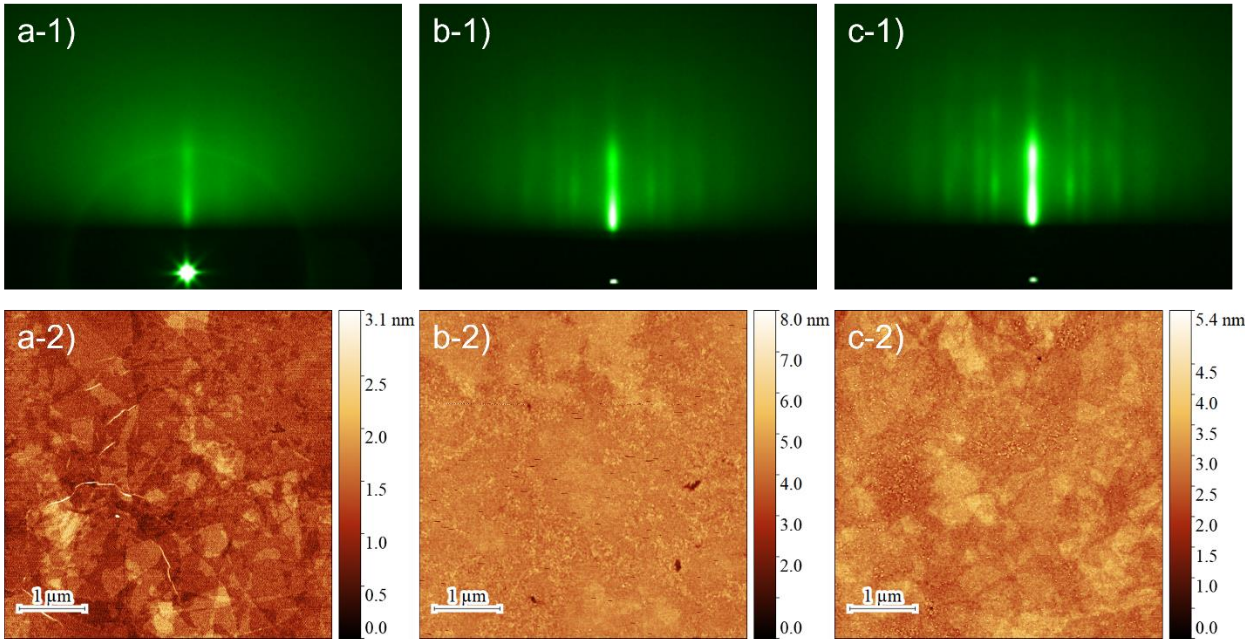

Figure S7. RHEED patterns (upper row) and AFM images (lower row) of STO/rGO/Si samples with STO thickness of (a) 3.9, (b) 39, and (c) 60 nm.

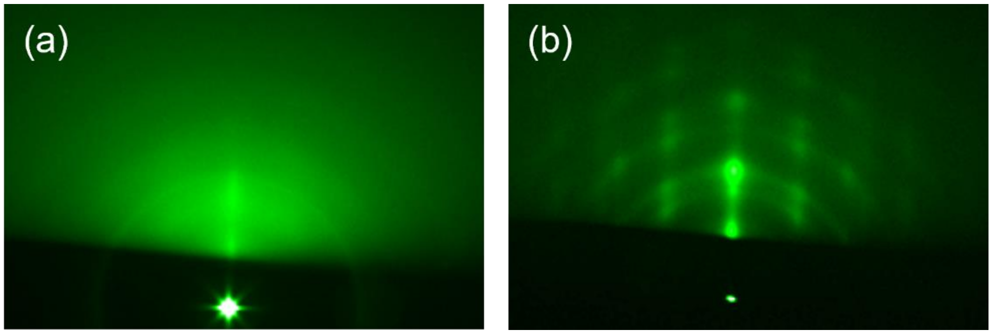

Figure S8. RHEED images of the Pt-covered sample (a) as-sputtered and (b) annealed at 500 °C for 30 min in vacuum. The spotty patterns in (b) are an indication of 3D Pt islands formation in contrary to diffuse background in (a) where the as-sputtered film was still amorphous.

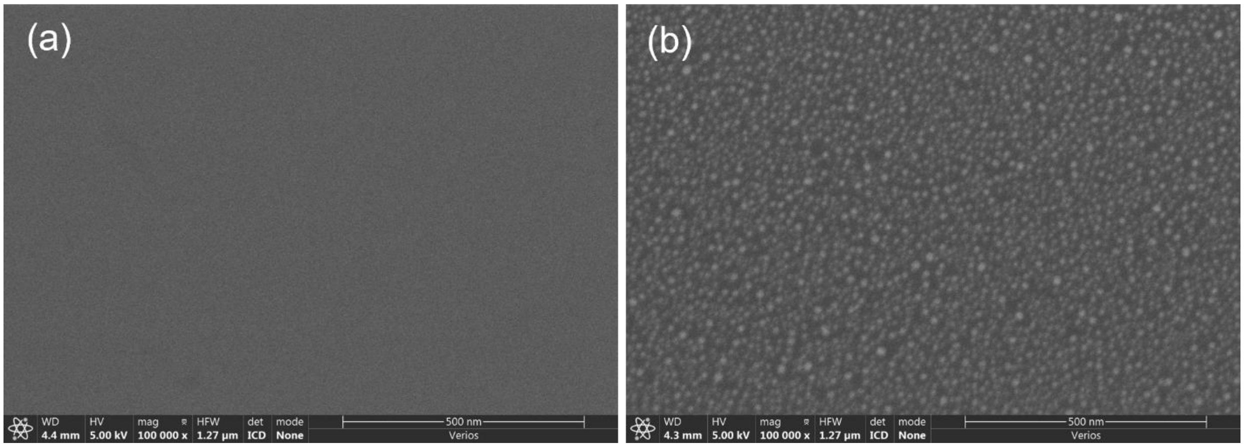

Figure S9. SEM images of the Pt-coated STO/rGO/Si sample (a) as-deposited and (b) after annealed at 500 °C for 30 min in vacuum.

Table S2. Comparison of our photocathode's performance with the literature.

Photocathode	Substrate	Electrolyte	Onset potential* (V_{RHE})	j (mA cm^{-2}) (@ 0 V_{RHE})	Ref.
$Pt/SrTiO_3/rGO/Si$	p-Si	1 M $HClO_4$	0.33	−5	This work
$MoS_2/TiO_2/Si$	pyramid-shape Si	0.5 M H_2SO_4	0.42	−0.24	S4
$TiO_2/NiMoO_{4-x}S_x/TiO_2/Si$	p-Si	0.1 M NaH_2PO_4	0.30	−0.5	S5
Ni/ferryhydrite/a-Si	p-i-n a-Si	1.0 M KOH	0.67	−15.6	S6
$Rh\text{-}P/MoS_2/TiO_2/p\text{-}Si$	p-Si	0.5 M H_2SO_4	0.43	−24.1	S7
Pt/graphene/pyramid Si	pyramid Si	1 M $HClO_4$	0.41	−32.5	S8
$C/\alpha\text{-}Fe_2O_3/Si$ NW	Si nanowires	1 M Na_2SO_4	−0.18	0	S9

* Defined as the position where current density reaches −0.1 mA cm^{-2}.

- **Half-cell solar-to-hydrogen (HC-STH) efficiency calculation**

The half-cell solar-to-hydrogen (HC-STH) of the photocathode represents the ability of the photocathode for converting water into hydrogen gas under solar light illumination, and can be calculated based on the following equation:[S1-S3]

$$\text{HC-STH} = \frac{|I_{\text{ph}}| \times \left(E_{\text{RHE}} - E_{\text{H}^+/\text{H}_2}\right)}{P_{\text{sun}}} \times 100\%,$$

where the $\left|I_{\text{ph}}\right|$ and E_{RHE} could be extracted from the $J-V$ plots (Figure 3a in the main text). $E_{\text{H}^+/\text{H}_2}$ is 0 V_{RHE}; P_{sun} is the incident light intensity. The results are shown in Figure S10.

We believe that the HC-STH efficiency can be further enhanced by improving the onset potential toward more positive region, which might be achieved using an alternative substrate such as buried junction n^+p-Si, where the photovoltage is defined within the built-in buried junction and not easily affected by the outer layer materials. Microstructure fabrication (Si nanorod, pyramid, etc.) could also be considered, as in this case the light harvesting and charge transport efficiency would be significantly improved. However, upon such kind of morphology it would be difficult to uniformly coat the GO layer using our developed spin-coating method. Hence, Langmuir-Schaefer method could be considered.

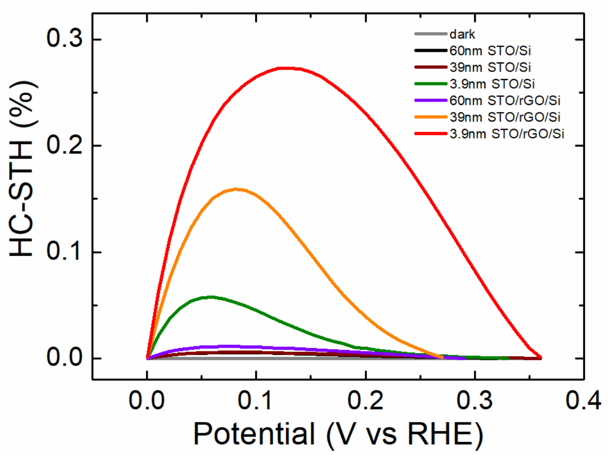

Figure S10. Half-cell solar-to-hydrogen conversion efficiency (HC-STH) derived from the $J-V$ plots in Figure 3a in the main text.

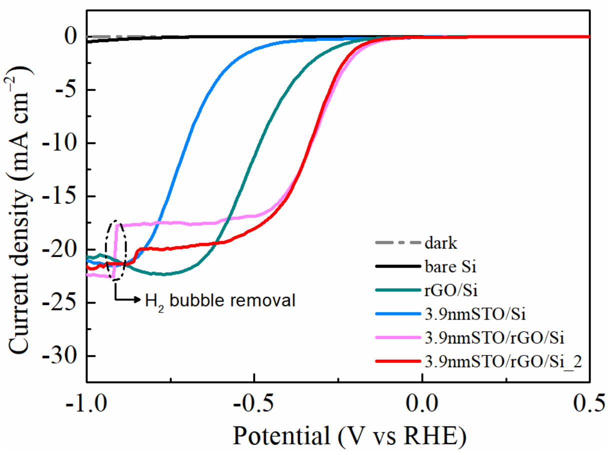

Figure S11. Polarization curves of samples without Pt co-catalyst under simulated solar light illumination at ~0.8 sun. The discontinuous photocurrent behavior of 3.9 nm STO/rGO/Si at −0.919 V (vs RHE) was caused by spontaneous H_2 bubble removal. The bubble blockage effect was confirmed by another LSV measurement shown in red curve where the saturation current was reached at larger value.

- **Discussion of charge separation and transfer in the STO/rGO/Si photocathode**

Based on the results and analyses, we propose a mechanism to illustrate the charge separation and transfer taking place in the STO/rGO/Si system upon solar light illumination (Figure S12). A metal-ion-semiconductor (MIS) Schottky junction formed in the rGO/silicate/p-Si heterostructure could favor the directional electron transport via tunneling through a thin amorphous silicate layer,[S10] given the metal-like rGO (4.22 eV) with smaller work function than Si (4.88 eV)[S11] and its redox potential lying close to E_{H^+/H_2} (−0.08 eV vs SHE, pH = 0).[S12] After the electron/hole pairs are photogenerated under solar illumination, wherein the intrinsic Fermi level is split into quasi-Fermi levels for electron and hole ($E_{F,n}$ and $E_{F,p}$, respectively) in a response to the deviation from thermodynamic equilibrium, the electrons could be quickly transported to rGO and then migrated through the conduction band of STO and ultimately, be exploited as a reducing source to converting water into hydrogen at the site of cocatalyst Pt nanoparticle. The well-documented near-zero conduction band offset between Si and STO is believed to be valid in our STO/rGO/Si system as well,[S13-S14] because from which a remarkably positive onset potential could be obtained. The band edge positions involved in the schematic diagram are incorporated from the literature reports.[S11-S15] It has to be mentioned that the observed photovoltage might be mainly contributed by the extent of Fermi level splitting in Si, which is related to the sample quality. The MIS Schottky junction (rGO/silicate/p-Si) could potentially give larger photovoltage than the pure insulator/semiconductor junction, where the photovoltage extraction barrier is larger in the latter.[S10] The quality of STO protection layer, on the other hand, is also likely to influence this extraction barrier. As discussed in the main text, numerous pinholes formed in the direct contacted STO/Si would expose Si to the electrolyte which in turn deteriorate the sample. The degraded

sample is inferior to the well-protected STO/rGO/Si in the sense of photovoltage and hence, the PEC performance.

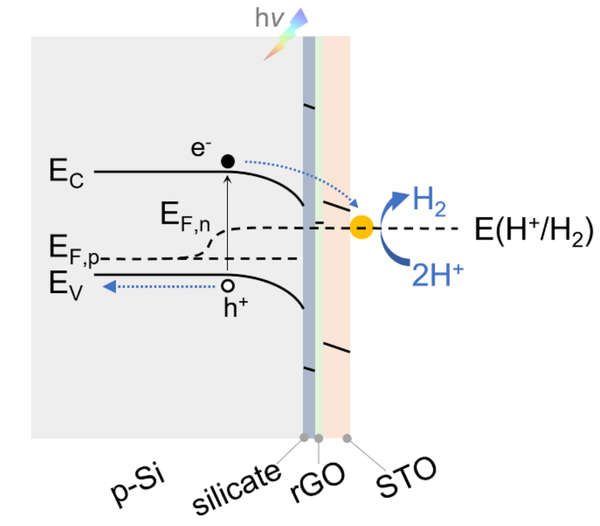

Figure S12. Schematic mechanism of charge separation and transfer of the STO/rGO/Si photocathode under solar illumination. E_c, E_v, $E_{F,p}$, and $E_{f,n}$, represent conduction band minimum, valence band maximum, quasi-Fermi level of hole, and quasi-Fermi level of electron, respectively.

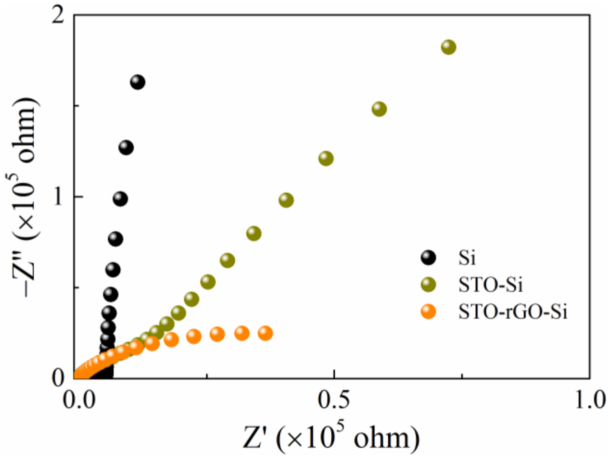

Figure S13. EIS measurements of samples without Pt co-catalyst under simulated solar light illumination at ~0.8 sun.

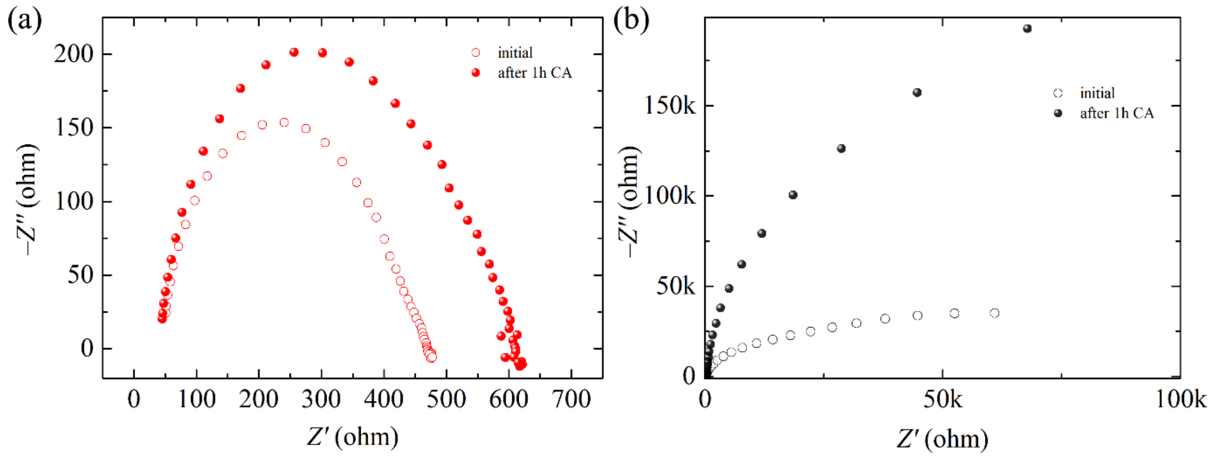

Figure S14. EIS results of (a) Pt/3.9nm STO/rGO/Si and (b) Pt/Si prior to (hollow circles) and after (solid circles) 1 hour stability measurements.

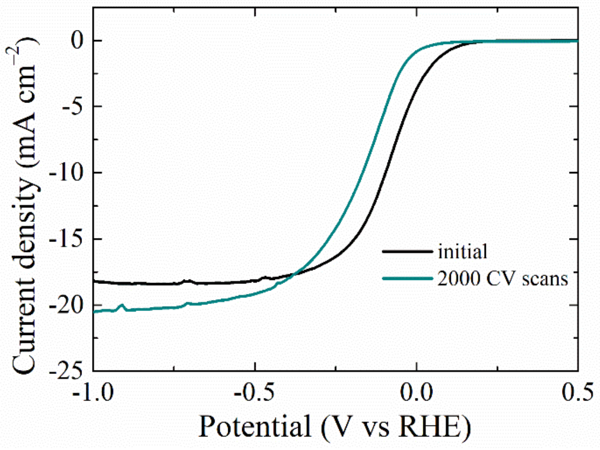

Figure S15. Polarization curves of Pt/STO/rGO/Si sample recorded before and after the stability tests with 2000 cyclic voltammetry (CV) scans (scan rate of 1 V/s) under simulated solar light illumination. During the CV scans, an ultrasonic probe sonicator was utilized to remove accumulated microbubbles from the sample surface (on/off periods of 2 seconds/1 minute).

Reference

S1. Kumagai, H.; Minegishi, T.; Sato, N.; Yamada, T.; Kubota, J.; Domen, K., Efficient solar hydrogen production from neutral electrolytes using surface-modified Cu(In,Ga)Se_2 photocathodes. *Journal of Materials Chemistry A* 2015, 3, 16, 8300-8307.

S2. Xiao, Y.; Fan, Z.; Nakabayashi, M.; Li, Q.; Zhou, L.; Wang, Q.; Li, C.; Shibata, N.; Domen, K.; Li, Y., Decoupling light absorption and carrier transport via heterogeneous doping in Ta_3N_5 thin film photoanode. *Nature Communications* 2022, 13, 1, 7769.

S3. Liang, X.; Wang, P.; Tong, F.; Liu, X.; Wang, C.; Wang, M.; Zhang, Q.; Wang, Z.; Liu, Y.; Zheng, Z.; Dai, Y.; Huang, B., Bias- free solar water splitting by tetragonal zircon $BiVO_4$ nanocrystal photocathode and monoclinic scheelite $BiVO_4$ nanoporous photoanode. *Advanced Functional Materials* 2021, 31, 8, 2008656.

S4. Li, X.; Li, Y.; Wang, H.; Miao, H.; Zhu, H.; Liu, X.; Lin, H.; Shi, G., Fabrication of a Three-Dimensional Bionic $Si/TiO_2/MoS_2$ Photoelectrode for Efficient Solar Water Splitting. *ACS Appl. Energy Mater.* 2021, 4, 1, 730–736.

S5. Wu, F.; Tian, W.; Cao, F.; Meng, L.; Li, L., Loading Amorphous $NiMoO_{4-x}S_x$ Nanosheet Cocatalyst to Improve Performance of *p*-Silicon Wafer Photocathode. *ACS Appl. Energy Mater.* 2018, 1, 3, 1286–1293.

S6. Zhang, D.; Du, M.; Wang, P.; Wang, H.; Shi, W.; Gao, Y.; Karuturi, S.; Catchpole, K.; Zhang, J.; Fan, F.; Shi, J.; Liu, S., Hole-Storage Enhanced a-Si Photocathodes for Efficient Hydrogen Production. *Angew. Chem. Int. Ed.* 2021, 60, 21, 11966–11972.

S7. Chen, Z.; Li, Y.; Wang, L.; Bu, Y.; Ao, J.-P., Development of a Bi-Compound Heterogeneous Cocatalyst Modified p-Si Photocathode for Boosting the Photoelectrochemical Water Splitting Performance. *J. Mater. Chem. A* 2021, 9, 14, 9157–9164.

S8. Ku, C. K.; Wu, P. H.; Chung, C. C.; Chen, C. C.; Tsai, K. J.; Chen, H. M.; Chang, Y. C.; Chuang, C. H.; Wei, C. Y.; Wen, C. Y.; Lin, T. Y.; Chen, H. L.; Wang, Y. S.; Lee, Z. Y.; Chang, J. R.; Luo, C. W.; Wang, D. Y.; Hwang, B. J.; Chen, C. W., Creation of 3D Textured Graphene/Si Schottky Junction Photocathode for Enhanced Photo-Electrochemical Efficiency and Stability. *Adv. Energy Mater.* 2019, 9, 29, 1901022.

S9. Qu, Y.; Li, F.; Zhang, P.; Zhao, L.; Liu, J.; Song, X.; Gao, L., Enhanced Photoelectrochemical Performance and Stability of Si Nanowire Photocathode with Deposition of Hematite and Carbon. *Appl. Surf. Sci.* 2019, 471, 528–536.

S10. Scheuermann, A. G.; Lawrence, J. P.; Kemp, K. W.; Ito, T.; Walsh, A.; Chidsey, C. E.; Hurley, P. K.; McIntyre, P. C., Design principles for maximizing photovoltage in metal-oxide-protected water-splitting photoanodes. *Nat. Mater.* 2016, 15, 1, 99–105.

S11. Maier, C. U.; Specht, M.; Bilger, G., Hydrogen evolution on platinum-coated p-silicon photocathodes. *Int. J. Hydrog.* 1996, 21, 10, 859–864.

S12. Xiang, Q.; Yu, J.; Jaroniec, M., Enhanced photocatalytic H_2-production activity of graphene-modified titaniananosheets. *Nanoscale* 2011, 3, 3670–3678.

S13. Chambers, S. A.; Liang, Y.; Yu, Z.; Droopad, R.; Ramdani, J.; Eisenbeiser, K., Band discontinuities at epitaxial $SrTiO_3$/Si(001) heterojunctions. *Appl. Phys. Lett.* 2000, 77, 11, 1662–1664.

S14. Ji, L.; McDaniel, M. D.; Wang, S.; Posadas, A. B.; Li, X.; Huang, H.; Lee, J. C.; Demkov, A. A.; Bard, A. J.; Ekerdt, J. G.; Yu, E. T., A silicon-based photocathode for water reduction with an epitaxial $SrTiO_3$ protection layer and a nanostructured catalyst. *Nat. Nanotechnol.* 2015, 10 ,1, 84–90.

S15. Longo, R. C.; Schewe, N.; Weidler, P. G.; Heissler, S.; Thissen, P., Synthesis of Silicates for High-Performance Oxide Semiconductors: Electronic Structure Analysis. *ACS Applied Electronic Materials 2020*, 3, 1, 299–308.
